# Clinical application of 3D-printed-step-bolus in post-total-mastectomy electron conformal therapy

**DOI:** 10.18632/oncotarget.12829

**Published:** 2016-10-23

**Authors:** Kwangwoo Park, Sungjin Park, Mi-Jin Jeon, Jinhyun Choi, Jun Won Kim, Yoon Jin Cho, Won-Seok Jang, Yo Sup Keum, Ik Jae Lee

**Affiliations:** ^1^ Department of Radiation Oncology, Gangnam Severance Hospital, College of Medicine, Yonsei University, Gangnam-gu, Seoul, Korea; ^2^ Department of Textiles, Fashion and Merchandising, College of Human Ecology, Seoul National University, Gwanak-gu, Seoul, Korea; ^3^ Texas A&M Health Science Center College of Medicine, Bryan, Texas, USA

**Keywords:** 3D-printed bolus, 3D printer, MRM, electron conformal therapy, dosimetery

## Abstract

The 3D-printed boluses were used during the radiation therapy of the chest wall in six patients with breast cancer after modified radical mastectomy (MRM). We measured the in-vivo skin doses while both conventional and 3D-printed boluses were placed on the chest wall and compared the mean doses delivered to the ipsilateral lung and the heart. The homogeneity and conformity of the dose distribution in the chest wall for both types of boluses were also evaluated. The uniformity index on the chest skin was improved when the 3D-printed boluses were used, with the overall average skin dose being closer to the prescribed one in the former case (-0.47% versus -4.43%). On comparing the dose-volume histogram (DVH), it was found that the 3D-printed boluses resulted in a reduction in the mean dose to the ipsilateral lung by up to 20%. The precision of dose delivery was improved by 3% with the 3D-printed boluses; in contrast, the conventional step bolus resulted in a precision level of 5%. In conclusion, the use of the 3D-printed boluses resulted in better dose homogeneity and conformity to the chest wall as well as the sparing of the normal organs, especially the lung. This suggested that their routine use on the chest wall as a therapeutic approach during post-mastectomy radiation therapy offers numerous advantages over conventional step boluses.

## INTRODUCTION

Electron conformal therapy has been used for treating superficial cancers and diseases for a long time, owing to the fact that it results in specific dose distribution. The electron beam delivers a uniform dose of radiation to the planning target volume (PTV) and has a sharp distal fall off. Furthermore, it reduces unnecessary irradiation to the underlying healthy normal tissue. However, inhomogeneous dose delivery in the target volume owing to irregularities in the skin surface and varying target depths can occur [1]. To compensate for the missing tissue, to ensure that the dose distribution conforms to the target volume, and to make the thickness of the chest wall uniform with a compensator, discontinuously step-structured boluses of various thicknesses have been employed. However, step boluses result in hot and cold spots in the dose distribution. To remedy this problem, boluses having continuously smooth surfaces and made of rubber [2] and wax and fabricated using computer-driven milling machines have been investigated [3, 4]. These efforts have successfully improved the conformity of the dose distribution by using appropriate dose calculation algorithms and customized boluses and have been extended to the treatment of head and neck tumors [5]. Furthermore, the technique of intensity modulation using wax boluses produced by computer-driven machines has also been introduced [6].

Recently, given the widespread availability of three-dimensional (3D) printers, Burleson et al. [7] have reported the benefits of such printers, which are lower operational and production costs. Moreover, Zou et al. [8] reported that 3D printers have the ability to fabricate compensators with much finer patterns. As this trends, Park et al. [9] used the 3D-printed bolus for Kimura's disease as a case report. However, the clinical and routinized application of boluses and compensators fabricated using 3D printers has not yet been attempted for the breast cancer patients. In this study, we employed 3D-printed boluses during the electron radiation therapy of the chest wall for six patients with breast cancer who had undergone mastectomy and compared the dose homogeneity, conformity, and normal organ sparing effects of the 3D-printed boluses with those of conventional step boluses.

## RESULTS

### Results of *in-vivo* measurements

**Figure 1:**
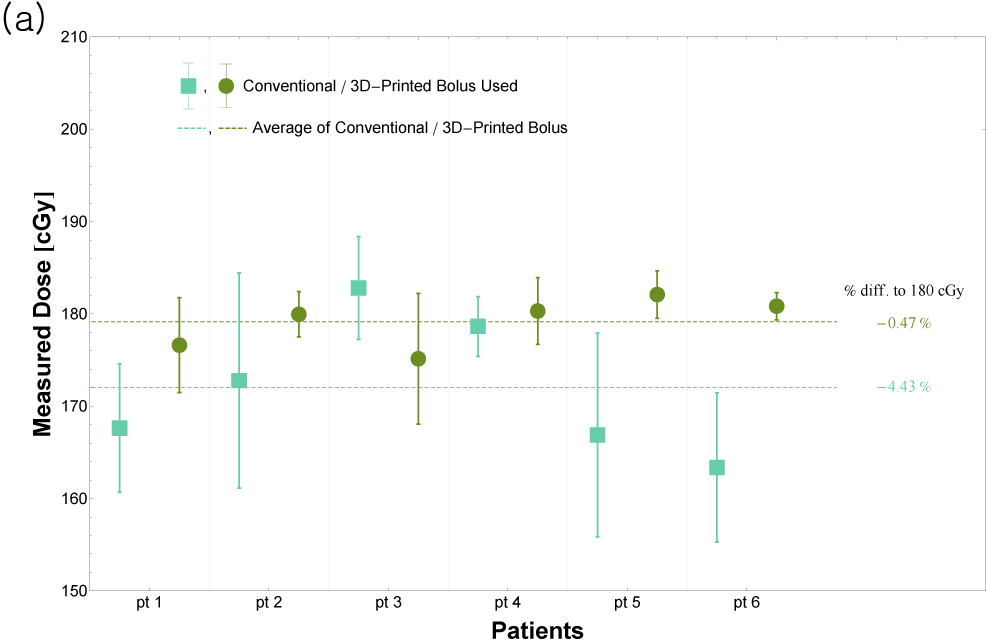

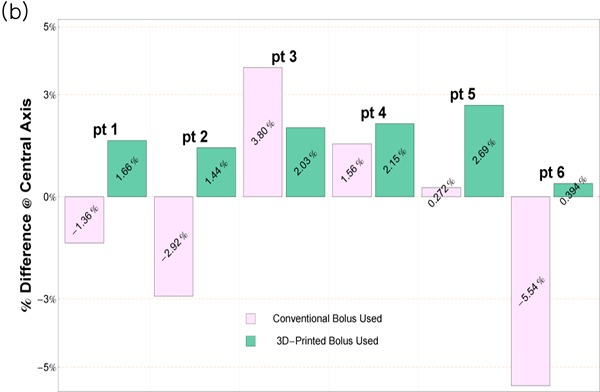
(**a**) Results of the in-vivo measurements of five skin doses for the six patients. Based on the values of the STD (error bars), it was concluded that the uniformity of the dose distribution on the chest skin improved with the use of the 3D-printed boluses. Further, the overall average value was closer to the prescribed dose in the case of the 3D-printed boluses. (**b**) Doses measured along the central axis. When the 3D-printed boluses were used, the precision improved to 3%; in contrast, that in the case of the conventional step bolus was 5%. A word “pt” stands for patient.

Figure 1 (a) shows the averages of the five skin doses for the six patients corresponding to the use of the conventional step bolus and the 3D-printed bolus. The overall average in the case of the 3D-printed boluses was closer to the prescribed dose (--0.47% percentage difference), while for the conventional step bolus, the overall average value was -4.43%. The standard deviations (STD) for the values measured at the five points were also shown as error bars; the STDs corresponding to the use of the 3D-printed boluses were much smaller than those to the use of the conventional step bolus. This showed that the uniformity of the dose distribution on the chest skin improved with the use of the 3D-printed boluses.

When the central doses were measured along the beam axis, the percentage differences compared to prescribed dose were 3% and 6% in the case of the 3D-printed boluses and the conventional step bolus, respectively (Figure 1 (b)).

### Results of plan comparison

**Figure 2:**
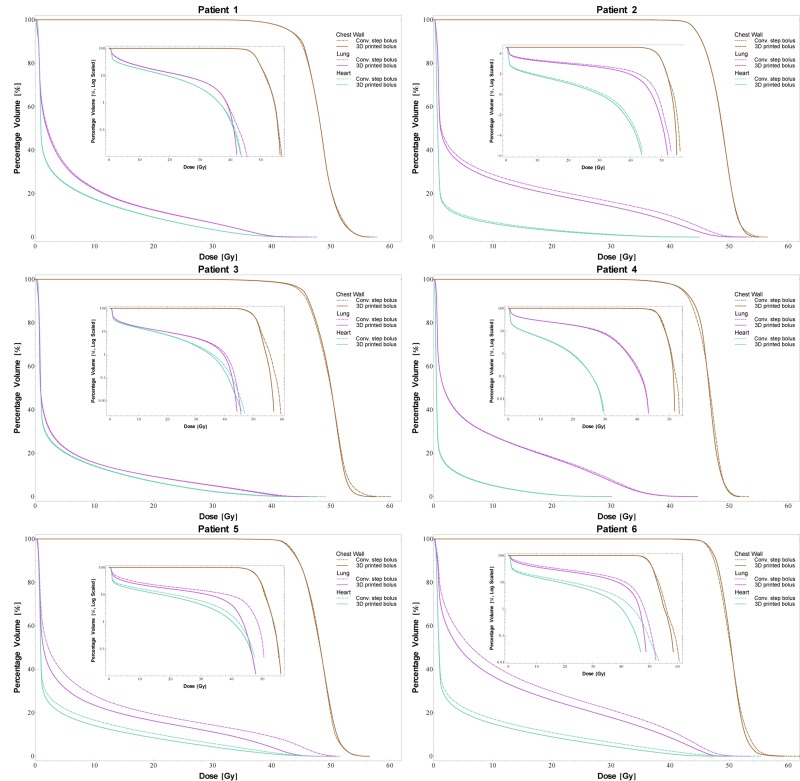
DVH curves for the six patients The inset plots are the log-scale DVH curves highlighting the differences between the two types of boluses for smaller differences in the percentage volume values. The use of the 3D-printed boluses resulted in lower doses to the normal organs (lungs and heart).

**Figure 3:**
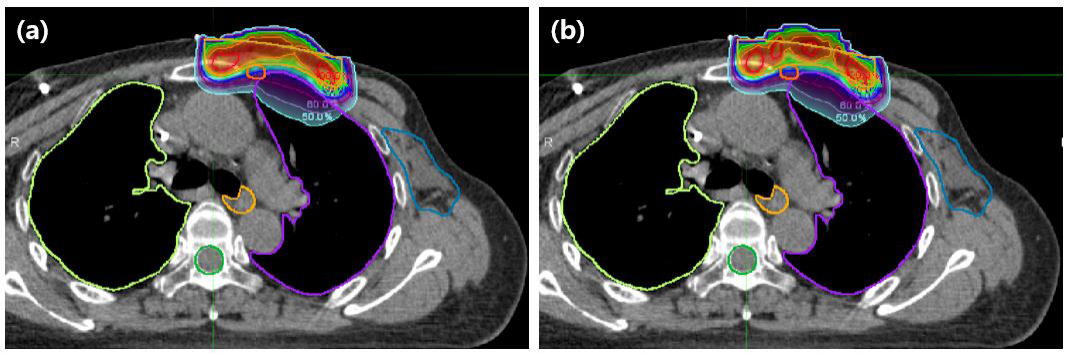
Example of dose distributions (**a**) The use of 3D printed bolus. (**b**) The use of conventional step bolus. In conventional step bolus, discontinuous shape of bolus showed several relatively hot and cold spots, while continuous and uniform depth of chest wall plus bolus reduced the hot and cold spots resulting in improving dose conformity and uniformity.

The DVH curves for all the patients are shown in Figure 2. The use of the 3D-printed boluses reduced the dose absorbed by the normal organs in most of the cases. The mean doses delivered to the ipsilateral lung and the heart, which are listed in Table 1, highlight the effectiveness of the 3D-printed boluses. The table also shows the standard deviations in the doses delivered to the chest wall, indicating the homogeneity of the dose distribution. In the case of patient 4, the mean dose to the heart increased with the use of the 3D-printed bolus, because the thickness of the 3D-printed bolus above the heart was lower than that of the conventional step bolus (the thickness of the 3D-printed bolus in this area was 2 mm while that of the conventional step bolus was 5 mm). However, the mean dose to the ipsilateral lung decreased when we used the 3D-printed boluses in all the patients. In patient 5, the mean dose to the ipsilateral lung was reduced by up to 20%. The homogeneity, described in terms of the standard deviation in the dose to the chest wall, also improved for most patients. The exception was patient 5; the homogeneity increased to 3.3%, which corresponded to a dose 0.1 Gy. For the conformity index (CI), all the patients exhibited improved results. For instance, the CI value of patient 6 improved to 45%, in contrast to the case when the conventional step bolus was used. In Figure 3, dose distributions of the cases for the use of 3D printed and conventional step boluses were showed respectively. In the case for the use of conventional step bolus, discontinuous shape of bolus showed several relatively hot and cold spots, while continuous and uniform depth of chest wall plus 3D-printed bolus reduced the hot and cold spots, which resulted in improving dose conformity and uniformity.

**Table 1:** The change in dosimetric parameters derived from DVH curve

Gy	*D*_mean_ (CW)	*D*_mean_ (Lung)		*D*_mean_ (Heart)		Conformity Index (Coverage of 90% isdose to CW)		Homogeneity *D*_std_ (CW)	
Bolus type	Both (normalized)	Conv. step	3D printed	Conv. step	3D printed	Conv. step	3D printed	Conv. step	3D printed
Patient 1	48.13	7.00	6.88 (-1.7%)	5.24	5.19(-1.0%)	0.65	0.70 (7.9%)	2.98	2.93 (-1.7%)
Patient 2	48.62	10.28	9.22 (-10.3%)	2.38	2.22(-6.8%)	0.86	0.86 (0.5%)	2.38	2.35 (-1.4%)
Patient 3	49.60	5.19	5.17 (-0.3%)	4.40	4.30(-2.2%)	0.78	0.92 (18.4%)	3.06	2.79 (-8.8%)
Patient 4	46.33	8.09	7.96 (-1.5%)	1.66	1.71(3.2%)	0.90	0.93 (2.7%)	2.43	2.30 (-5.3%)
Patient 5	48.00	10.02	8.02 (-20%)	5.44	4.65(-14%)	0.65	0.71 (9.5%)	2.53	2.62 (3.3%)
Patient 6	50.31	14.13	12.17 (-14%)	5.67	4.73(-17%)	0.53	0.76 (45.0%)	2.29	2.04 (-11%)
Mean	48.50	9.15	8.24	4.13	3.80	0.72	0.81	2.61	2.51
Median	48.38	9.06	7.99	4.82	4.48	0.72	0.81	2.48	2.49

All parameters are normalized based on the condition of the same mean dose on chest wall.

All unit is Gy except for unitless conformity index.

The number in parenthesis is percentage difference compared to conventional step bolus

Conv. and CW stand for “conventional” and “chest wall” respectively.

## DISCUSSION

In this study, we used individually customized boluses, which were designed to compensate for the differences in the chest wall thickness and to reduce the irradiation dose delivered to the lungs and the heart during the irradiation of the chest wall of post-mastectomy breast cancer patients. While using the individually customized boluses with the reverse hockey stick method, the normal tissue complication probability of the ipsilateral lung was reduced by approximately 24.5–40.5%; this was in line with the decrease in the rate of radiation pneumonitis development [10].

The use of the 3D-printed boluses can be compared to the use of boluses fabricated from resin-impregnated wax using a computer-driven milling machine [3]. Thus, it was worthwhile to compare the two bolus types. According to Low et al. [3], the manufacturing precision of conventional boluses is determined by the tool path spacing and the diameter of the ball-end mill bit, which were 2 mm and 4 mm, respectively. On the other hand, the precision of the 3D printing system used in this study was determined by both the printing layer thickness and the nozzle diameter, which were 0.5 mm and 0.4 mm, respectively. Therefore, the manufacturing precision of the 3D-printed boluses was higher, thereby resulting in more precise dose delivery. The physical densities of wax and polyactic acid (PLA, material used in 3D-printing) are 0.92 and 1.19, respectively, which are close to that of soft tissue. However, acrylonitrile butadiene styrene (ABS) is used preferentially as a tissue-equivalent material. Thus, it is necessary to improve the properties of ABS so that it can be used with 3D printing. This would entail fixing issues such as the bending and warping of ABS during 3D printing.

The results of this study confirmed that the dosimetric parameters improved with the use of the 3D-printed boluses instead of the conventional step bolus. This included improvements in the precision of the central dose, dose homogeneity, and the CI value. However, in some cases (e.g., patient 4), the mean dose to the heart was lower in the case of the conventional step bolus, because the minimum thickness of the conventional step bolus was 5 mm, while it was 2 mm in the case of the 3D-printed bolus. In order to overcome problems such as that encountered in the case of patient 4, the design of the 3D-printed boluses should be modified using a computer algorithm. For example, a thickness constraint could be applied to reduce the dose absorbed by the normal organs. The computer-aided design of 3D-printed boluses could allow for more precise shape control and improve the dosimetric parameters. As suggested by Su [11], the computer-aided design process could be expanded to the bolus-based modulation of electron radiation therapy (MERT). The anatomic inhomogeneities encountered hinder precise dose delivery, an important factor to consider in the development of the algorithms for MERT.

The technique used in this study could easily be extended to the treatment of other superficial lesions. A 3D image of the area to be treated can be acquired from imaging sources such as MRI, CT, and optical 3D imaging scans for both electron and photon beam therapy. Even though the development of materials that can be considered tissue equivalent as well as reductions in the printing time are still necessary, the dosimetric results obtained in this study confirmed that the technique is suitable for clinical use.

In conclusion, the main purpose of this study was to confirm whether 3D-printed boluses are better suited instead of conventional step boluses for use during electron conformal therapy. Based on in-vivo measurements, it was found that the 3D-printed boluses improved the precision of the dose absorbed by the chest wall to 3%; in contrast, the use of the conventional step bolus resulted in a dose uncertainty of up to 6%. Furthermore, the homogeneity of the dose distribution both on the surface and within the chest wall was improved. In addition, the dose absorbed by the normal organs was reduced by up to 20%. From these results, it can be concluded that there are a number of advantages of employing 3D-printed boluses for routine electron conformal therapy following MRM. Lastly, we have provided the details of the procedure for designing and creating 3D-printed boluses in detail for other users without having to take the trial-and-error approach.

## MATERIALS AND METHODS

### 1. Fabrication of 3D-printed customized boluses

**Figure 4:**
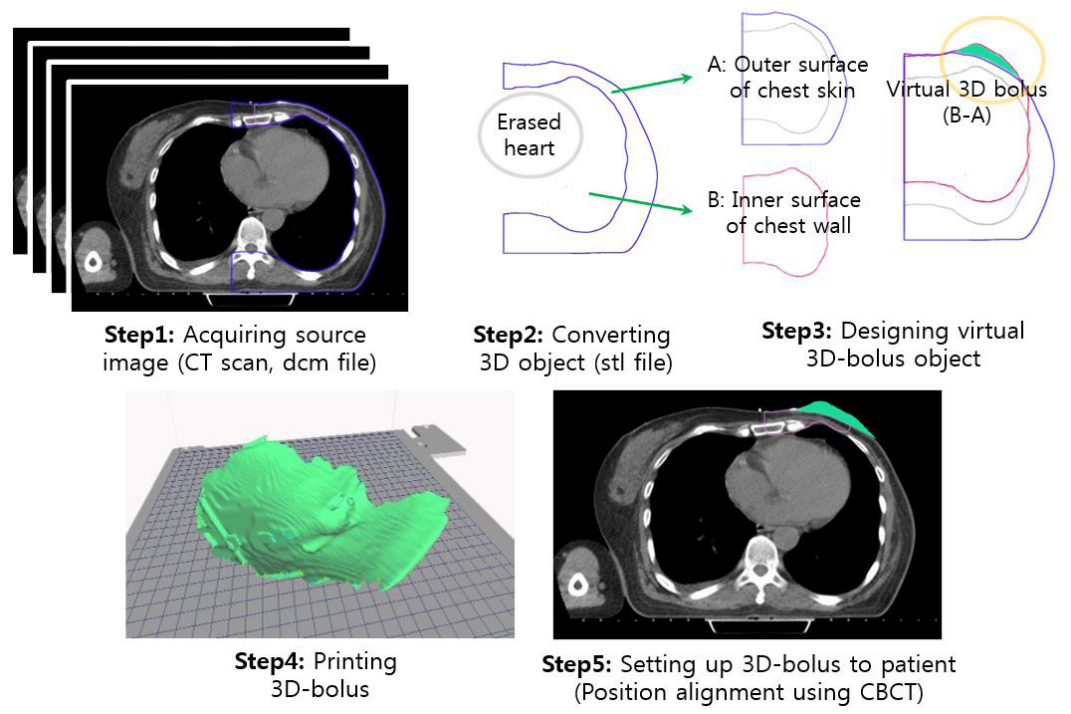
Schematic description of the procedures involves, which ranged from CT image acquisition to the placement of the 3D-printed bolus on the patient

Several studies have confirmed the advantages of electron conformal therapy performed using customized 3D-printed electron boluses and compensators over that performed with conventional ones [7, 8, 11, 12]. However, since these studies used the 3D-printed boluses only on phantoms and did not provide details of the procedures used, there remained a lack of information regarding their use with actual patients. Thus, in this paper, we describe the procedures used in detail so that they can be used by other users as well, eliminating the need for a trial-and-error approach. The five steps involved are outlined in Figure 4 and are described below.

#### Step 1: Acquiring source image

The image data for each patient as the DICOM (the digital imaging and communications in medicine) file format was acquired during computed tomography (CT) simulation. A CT slice was collected every 3 mm and reconstructed as a 1-mm-spaced slice. This source image was modified as required. As an example, to ensure clean and sharp images of the inner surface of the chest wall, it was sometimes necessary to erase the adjacent organs, such as the heart, from the collected images. In addition, to allow for precise placement of the bolus on the patient's chest, position markers were added to the images. The modifications to the CT images were performed using the MIM Maestro software (version 6.1, MIM Software Inc., USA).

#### Step 2: Converting source images into 3D object

The acquired source images were used to create virtual 3D-printable boluses. This was done using two applications: *3D* Slicerwww.slicer.organd *Blender* www.blender.org. These applications are open source and free to use in any institution or clinic. The *3D Slicer* was used to convert the patient's CT data into the data (in the stereolithography (STL) file format) that could be read by 3D printer applications and to define the region to be used to form the bolus. An initial 3D bolus object was created in *3D Slicer* having the surface information in terms of triangular meshes, the number of which determines the file size as well as the smoothness of the object surface.

#### Step3: Designing virtual 3D bolus

The initial 3D virtual object defined in the STL file was imported into *Blender*, in order to be able to control the mesh and modify the shape of the bolus. The converted raw 3D object from *3D Slicer* was refined by eliminating any defective meshes. Since there were a number of meshes, it was not possible to find and remove the defected ones manually. Thus, the cleaning and fixing of the meshes was done by forming a new surface; this, in turn, was accomplished by creating a sphere and using it as a new surface. The initial 3D object was wrapped within the sphere using the built-in “shrink-wrap” function in *Blender*. This new surface, which was identical to the surface of the initial 3D object, replaced the original surface.

The shape of a 3D-printable bolus should be such that it has the same contours as the lung-sided and skin-sided chest walls of the patient. The upper surface of the 3D-printable bolus should conform to the lung-sided surface of the chest wall, while the lower surface should conform to the skin-sided surface to compensate missing tissue for the uniform thickness of chest wall as shown in Figure 4. To design the 3D virtual boluses, two objects (A and B in Figure 4) were extracted from the raw 3D object; these conformed to the chest skin (body-shaped object, A in Figure 4) and lung-sided surface of the chest wall (lung-shaped object, B in Figure 4), respectively. The final bolus was obtained from the difference in these two objects. In other words, the upper surface of 3D-printed bolus extracted from the lung shaped object (B in Figure 4), and the lower surface from the chest skin (A in Figure 4).

#### Step 4: Printing actual 3D bolus

After the completion of the bolus design process, the boluses were fabricated with a 3D printer using PLA filament, whose physical density (p) was 1.19 kg/m^3^ and different from that of human tissue. We did not use ABS as the fabrication material even though it has a density similar to that of tissue, because ABS filaments more than 5cm in size tended to bend during the cooling process. To calculate the dose in TPS, the 3D-printed bolus in the CT scan was overrided as the density of 1.19. We used a CubeX^®^ (3D systems, USA) as the printing hardware and KISSlicerwww.kisslicer.comas the software; this software is available under an open-source license. The boluses were printed used the following parameters; speed of 30 mm/s, layer thickness of 0.5 mm, and infill ratio of 100%. The other parameters in *KISSlicer* were determined after careful calibration; for this purpose, we repeatedly printed cubes with a volume of 3 cm^3^. These printing parameters were chosen to reduce the time to print. The average printing time for the six 3D-printed boluses was less than 6 hours.

#### Step 5: Placing bolus on patient

To place the 3D-printed boluses on the patients with precision, cone-beam CT (CBCT) images were acquired, in order to align the position of the bolus by comparing it with the planned CT image. Further, a gel (Progel^®^; p = 1.02 kg/m^3^) that is used for acquiring ultrasonic images was employed for eliminating any possible air gaps between the patient skin and the bolus. The alignment of the 3D-printed boluses using CBCT was performed only during the first treatment fraction.

### 2. Clinical applications and in-vivo measurements

#### Patient characteristics and prescribed dose

The 3D-printed boluses were used with six patients, as shown in Table 2, who had been prescribed radiation therapy following MRM. Radiation was given once per day at doses of 1.8Gy per fraction (28 fractions, 50.4Gy) on the skin along the central axis. The treatment plan was implemented using the reverse hockey stick technique [13] and performed by the treatment planning system (TPS), RayStation (RaySearch Laboratories, USA) based on CT scan acquired from the CT simulator, Somatom (Siemens, Germany). The delineation of PTV and normal structures followed RTOG and ESTRO guidelines [14]. All patients were treated by the machine, Elekta Versa HD which has electron modalities of 6, 9, 12, and 15MeV. This Institutional Review Board of the Gangnam Severance Hospital, Korea (IRB No. 3-2015-0325) approved this retrospective study in accordance with ethical guidelines and the Declaration of Helsinki.

**Table 2:** Patient characteristics

Patient	Age	pathology	T stage	N stage	Tumor size (diameter)	Site
Pt 1	52	IDC	3	3	5.5cm	LUO (Left)
Pt 2	54	IDC	2	2	2.5cm	RUC (Right)
Pt 3	65	IDC	2	2	2.5cm	LOC (Left)
Pt 4	57	ILC	3	2	6.0cm	ROC (Right)
Pt 5	58	IDC	4	2	7.0cm	All quadrant (Left)
Pt 6	59	IDC	2	2	3.9cm	LUO (Left)

#### *In-Vivo* measurements of skin dose

**Figure 5:**
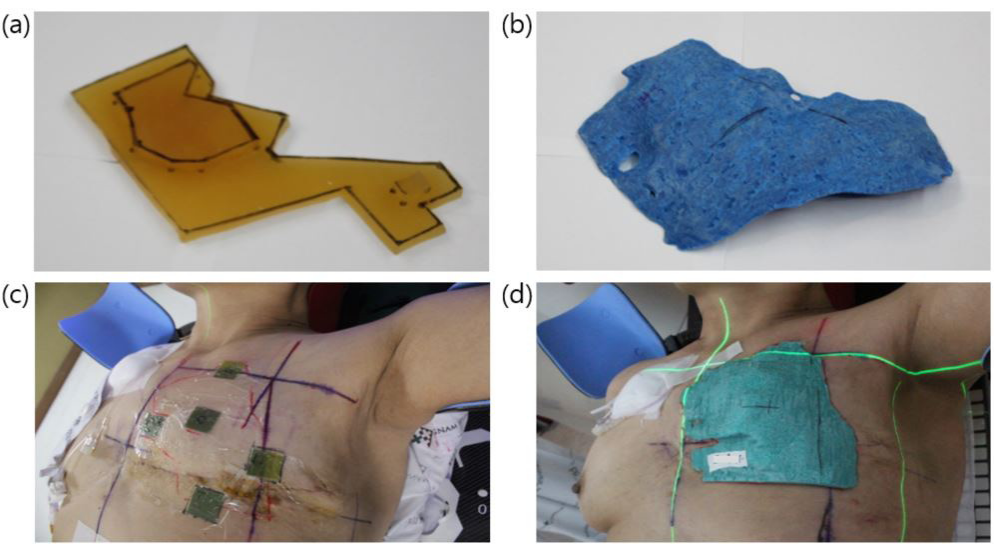
(**a**) The conventional step bolus and (**b**) a 3D-printed bolus. The conventional step bolus was made of two 5-mm-thick Superflab® boluses. And examples of in-vivo measurements: (**c**) the five measurement points on a patient's chest and (**d**) the placement of a 3D-printed bolus on patient 6

For the measurements of skin dose, Gafchromic films, EBT3 (Radiation Products Design, Inc., USA) were used to verify whether the dose absorbed by the chest skin was in keeping with the desired value. The measurements were performed using the two kinds of boluses, as shown in Figure 5 (a) and (b): a conventional step bolus at the first fraction and a 3D-printed one for the other fractions. A dosimetric comparison of the two types of boluses helped validate the applicability of the 3D-printed boluses. The measurements were made at five points, including at the position of the central axis, as shown in Figure 5 (c).

#### Plan comparison

In addition to the in-vivo measurements, the two plans used with the conventional step bolus and the 3D-printed bolus were compared. The two plans had the same dose prescribed and were normalized to have the same mean dose of the chest wall, in order to simulate two independent plans. We calculated the mean doses for both the ipsilateral lung and the heart from the normalized DVH curves and compared the homogeneities of the calculated doses for the chest wall on being irradiated by the electron beam; these were also determined from the DVH curves [15]. The differential DVH curve can be obtained from the derivative of the cumulative DVH. If the differential DVH is expressed as a function of the dose, *v*(*D*), the mean dose, *D*_mean_, and the standard deviation in the dose in the region of interest, then, *D*_std_, can be evaluated as follows:

$$\begin{array}{l} D_{mean}=\frac{\int_{0}^{D_{\max}}D\text{·}v\left(D\right)dD}{\int_{0}^{D_{\max}}v\left(D\right)dD}\\ D_{std}=\sqrt{\frac{\int_{0}^{D_{\max}}{\left(D-D_{mean}\right)}^{2}\text{·}v\left(D\right)dD}{\int_{0}^{D_{\max}}v\left(D\right)dD}} \end{array}$$ (1)

The conformity index (CI) is also an important dosimetric parameter and gives an idea of how well the PTV is covered by the radiation field. We calculated the CI value using the method suggested by Lomax and Scheib [16].

$$CI=\frac{TV_{RI}}{V_{RI}}$$ (2)

where V_RI_ is the volume of the reference isodose (90%), and TV_RI_ is the target volume covered by the reference isodose.
